# The proteoglycan-like domain of carbonic anhydrase IX mediates non-catalytic facilitation of lactate transport in cancer cells

**DOI:** 10.18632/oncotarget.25371

**Published:** 2018-06-15

**Authors:** Samantha Ames, Silvia Pastorekova, Holger M. Becker

**Affiliations:** ^1^ Division of General Zoology, Department of Biology, University of Kaiserslautern, Kaiserslautern, Germany; ^2^ Department of Tumor Biology, Institute of Virology, Biomedical Research Center, Slovak Academy of Sciences, Bratislava, Slovak Republic; ^3^ Institute of Physiological Chemistry, University of Veterinary Medicine Hannover, Hannover, Germany

**Keywords:** monocarboxylate transporter, proton antenna, hypoxia, breast cancer, Xenopus oocyte

## Abstract

Highly glycolytic tumor cells release vast amounts of lactate and protons via monocarboxylate transporters (MCTs), which exacerbate extracellular acidification and support the formation of a hostile environment. Transport activity of MCTs can be facilitated by non-catalytic interaction with carbonic anhydrase IX (CAIX), the expression of which has been shown to be upregulated under hypoxia. We have now studied the mechanisms that enable CAIX-mediated facilitation of proton-coupled lactate transport in breast cancer cells and *Xenopus* oocytes. Our results indicate that the proteoglycan like (PG) domain of CAIX could function as ‘proton antenna’ to facilitate MCT transport activity. Truncation of the PG domain and application of a PG-binding antibody significantly reduced proton-coupled lactate transport in MCT-expressing oocytes and hypoxic breast cancer cells, respectively. Furthermore, application of the PG-binding antibody reduced proliferation and migration of hypoxic cancer cells, suggesting that facilitation of proton-coupled lactate flux by the CAIX PG domain contributes to cancer cell survival under hypoxic conditions.

## INTRODUCTION

The most aggressive and invasive tumor cells rely on extensive glycolysis to meet their large demand for energy and biosynthetic precursors [1–4]. The increase in glycolytic activity is often triggered by hypoxia, which derives from high cell density, accompanied by insufficient vascularization [5–7]. The increase in glycolysis leads to vast production of lactate and protons that have to be removed from the cell to prevent acidosis, which, among other effects, would result in inhibition of glycolysis. Efflux of lactate from cancer cells is primarily mediated by the monocarboxylate transporters MCT1 and MCT4, both of which carry lactate in co-transport with H^+^ [7–10]. MCT-mediated H^+^ efflux exacerbates extracellular acidification and supports the formation of a hostile environment where cancer cells, that have adapted to these conditions, can outcompete normal cells, which further enhances tumor progression [4, 7, 11–13].

Another key protein in tumor acid/base regulation is the hypoxia-regulated carbonic anhydrase CAIX, which catalyzes the reversible hydration of CO_2_ to HCO_3_^-^ + H^+^. CAIX, the expression of which is usually linked to poor prognosis, is tethered to the membrane via a transmembrane domain, with the catalytic center facing the extracellular site. Like other fast carbonic anhydrases, CAIX is equipped with an intramolecular proton shuttle for rapid exchange of H^+^ between the catalytic center and the surrounding bulk solution. CAIX features a 59 amino acids long proteoglycan-like (PG) domain that is unique to CAIX among the CA family [14]. The PG domain of human CAIX contains 18 glutamate and 8 aspartate residues. These 26 COOH side chains have been suggested to function as an intramolecular proton buffer, which could support CAIX catalytic activity when operating in an acidic environment [15]. Furthermore, the PG domain seems to be involved in cell-cell adhesion and intercellular communication, since both PG deletion and treatment with the PG-binding antibody M75 reduces adhesion and spreading of cancer cells [16, 17].

We could recently show that CAIX enhances proton-coupled lactate transport in hypoxic MCF-7 breast cancer cells by a mechanism that is independent from the enzyme's catalytic activity [18]. Measurements in *Xenopus* oocytes revealed that CAIX facilitates transport activity of MCT1 and MCT4, presumably by functioning as a ‘proton antenna’ for the transporter [18]. Protonatable residues with overlapping Coulomb cages could form proton-attractive domains and could share a proton at a very fast rate, exceeding the upper limit of diffusion-controlled reactions [19, 20]. When these residues are located in proteins or lipid head groups at the plasma membrane, they can collect protons from the solution and direct them to the entrance of a proton-transfer pathway of a membrane-anchored protein, a phenomenon termed ‘proton-collecting antenna’ [19, 21]. The need for such a ‘proton antenna’ is based on the observation that H^+^ cotransporters, such as MCTs, extract H^+^ from the surrounding area at rates well above the capacity for simple diffusion to replenish their immediate vicinity. Therefore, the transporter must exchange H^+^ with protonatable sites at the plasma membrane, which could function as a ‘proton antenna’ for the transporter [22].

In the present study we investigated the role of the PG domain in CAIX-mediated facilitation of lactate transport. Our results suggest that the CAIX PG domain could function as a ‘proton antenna’ for MCT1 and MCT4, which mediates the rapid exchange of protons between the transporter pore and surrounding protonatable residues to drive proton-coupled lactate flux in hypoxic cancer cells.

## RESULTS

### CAIX-mediated facilitation of lactate transport requires the enzyme's PG domain

**Figure 1:**
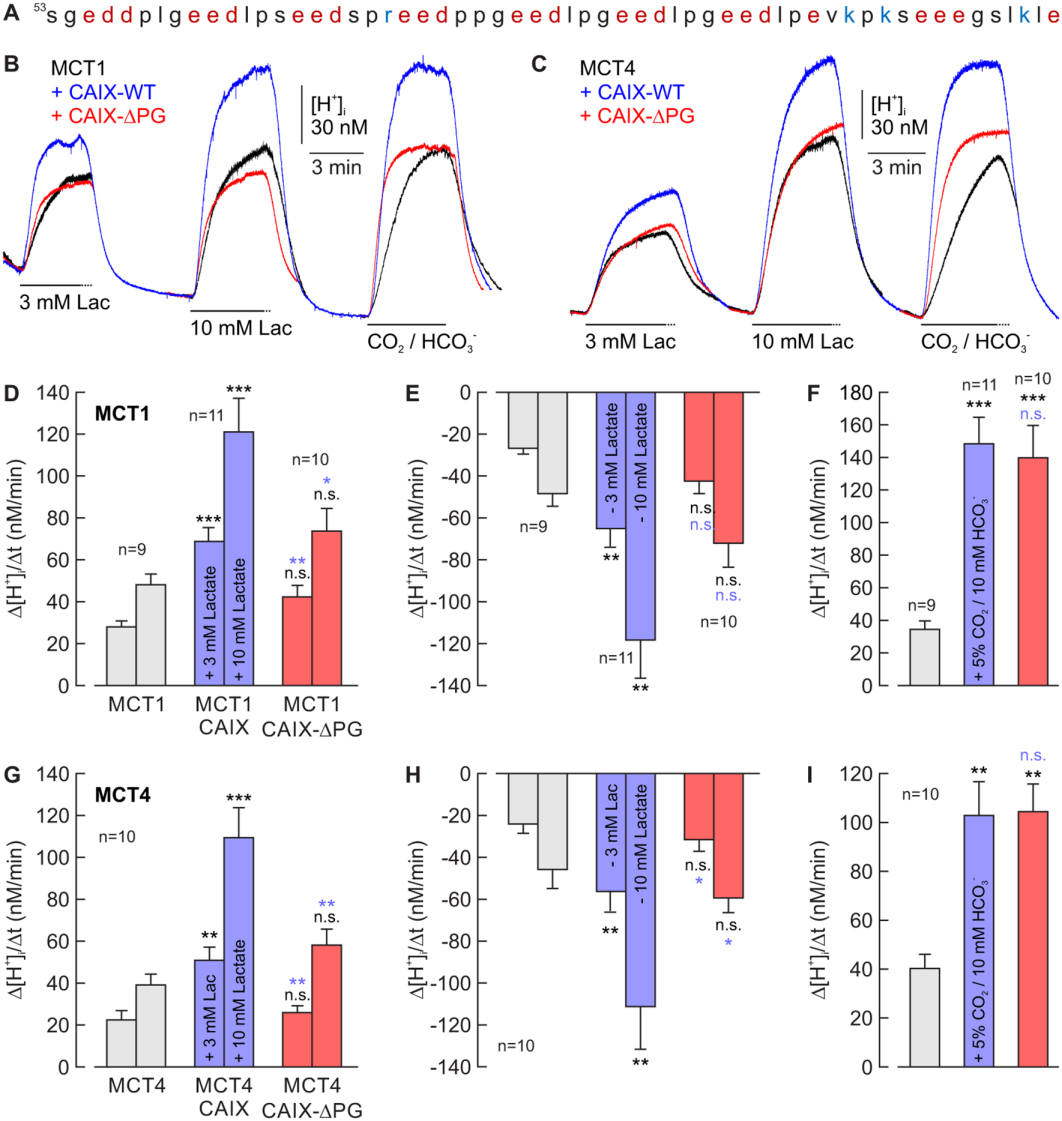
The PG domain of CAIX is involved in facilitation of MCT1/4 transport activity **(A)** Amino acid sequence of the human CAIX proteoglycan-like domain. Negatively charged amino acids are labelled in red, positively charged amino acids are labelled in blue. **(B, C)** Original recordings of the change in intracellular H^+^ concentration ([H^+^]_i_) in oocytes expressing (B) MCT1 or (C) MCT4 (black trace), MCT1/4 + CAIX-WT (blue trace), and MCT1/4 + CAIX-ΔPG (red trace), respectively, during application of 3 and 10 mM of lactate and 5% CO_2_ / 10 mM HCO_3_^-^. **(D-I)** Rate of change in intracellular H^+^ concentration (Δ[H^+^]/Δt) as induced by application (D, G) and removal (E, H) of 3 and 10 mM lactate, and application of 5% CO_2_ / 10 mM HCO_3_^-^ (F, I), respectively, in oocytes expressing MCT1/4 (gray), MCT1/4+CAIX-WT (blue), and MCT1+CAII-ΔPG (red), respectively. Data are represented as mean + SEM. Significance in differences was tested with ANOVA, followed by means comparison. The black significance indicators above the bars for MCT1/4+CAIX/CAIX-ΔPG-coexpressing oocytes refer to the values from oocytes expressing MCT1/4 alone. The blue significance indicators above the bars for MCT1/4+CAIX-ΔPG-coexpressing oocytes refer to the values from oocytes coexpressing MCT1/4 + CAIX.

We have recently shown that extracellular CAIX can facilitate transport activity of MCT1 and MCT4 in hypoxic breast cancer cells and *Xenopus* oocytes [18]. Facilitation of lactate transport was found to be independent of the enzyme's catalytic activity, which led to the conclusion that CAIX could function as an extracellular ‘proton antenna’ for MCTs. To investigate whether the PG domain of CAIX, which contains a high proportion of charged amino acids (Figure 1A) and might therefore serve as ‘proton antenna’, is involved in the facilitation of MCT transport activity we coexpressed MCT1 and MCT4, respectively, together with CAIX-WT or a CAIX mutant lacking the PG domain (CAIX-ΔPG) in *Xenopus* oocytes. MCT transport activity was monitored by measuring changes in intracellular proton concentration ([H^+^]_i_) during application and removal of lactate (Figure 1B, 1C). CAIX catalytic activity was determined by the rate of change in [H^+^]_i_ (Δ[H^+^]_i_/Δt) during application of CO_2_/HCO_3_^-^. Coexpression with CAIX-WT resulted in a more than twofold increase in transport activity of MCT1 and MCT4, as measured by the increase in Δ[H^+^]_i_/Δt during application (Figure 1D, 1G) and withdrawal of lactate (Figure 1E, 1H). In contrast to that, coexpression of MCT1 and MCT4 with CAIX-ΔPG resulted only in a slight increase in MCT transport activity, which was significantly reduced as compared to MCT1/4 + CAIX-WT. While the CAIX PG domain is required to facilitate MCT transport activity, catalytic activity of CAIX is not augmented by the PG domain in intact oocytes, since the rate of CO_2_-induced acidification remained unaltered between CAIX-WT- and CAIX-ΔPG-expressing oocytes (Figure 1F, 1I).

**Figure 2:**
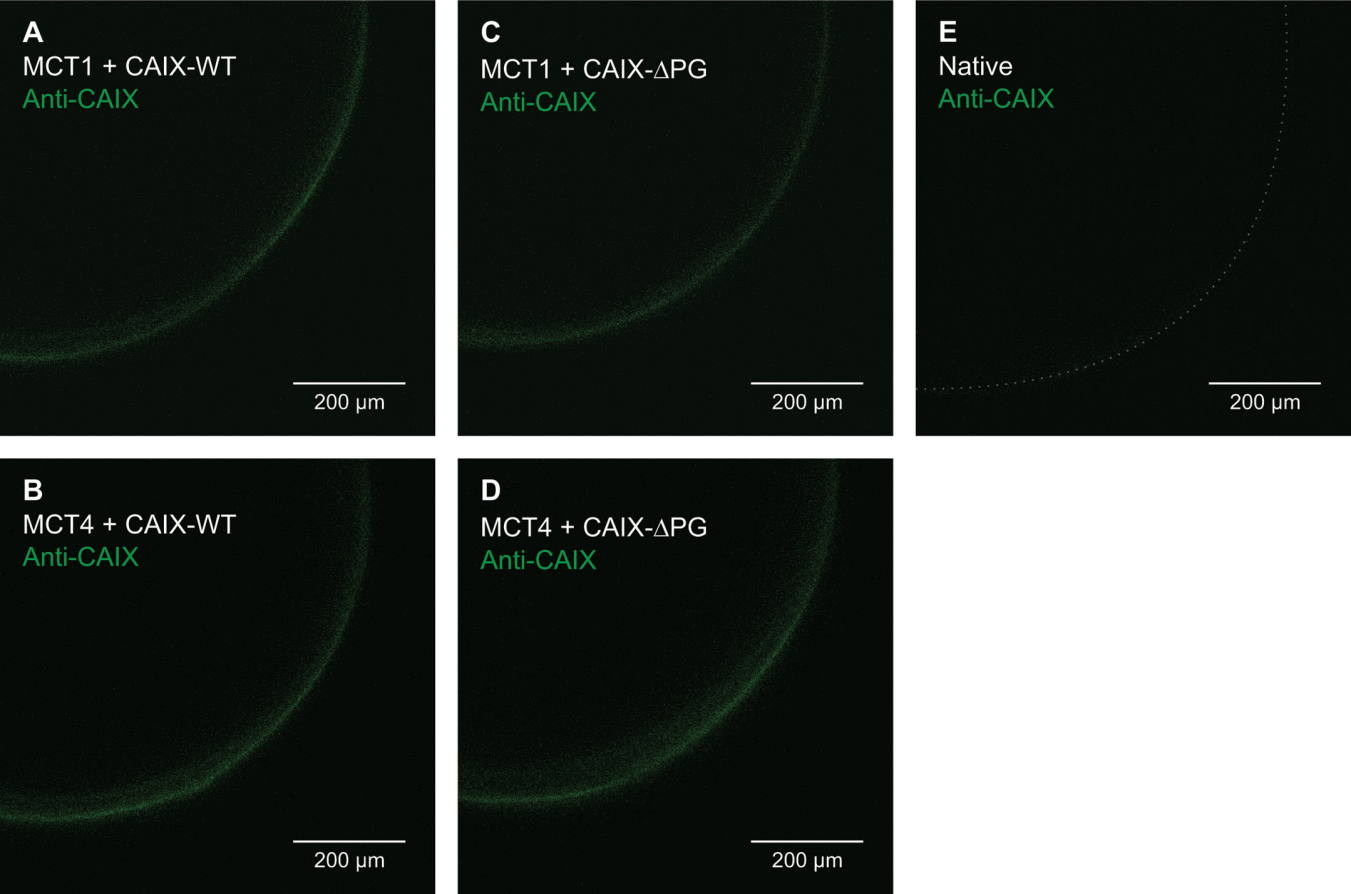
Localization of CAIX-WT and CAIX-ΔPG in *Xenopus* oocytes Antibody staining of fixed and permeabilized oocytes, expressing MCT1+CAIX-WT **(A)**, MCT4+CAIX-WT **(B)**, MCT1+CAIX-ΔPG **(C)**, MCT4+CAIX-ΔPG **(D)**, and a native oocyte as control **(E)**. CAIX was labeled with an antibody, mapping against the C-terminal region of CAIX. Pictures were taken with a confocal laser scanning microscope.

The almost identical rate of acidification during application of CO_2_/HCO_3_^-^ (Figure 1F, 1I) indicates that CAIX-WT and CAIX-ΔPG are expressed at equal levels in *Xenopus* oocytes. To investigate whether localization of CAIX in the plasma membrane does also remain unchanged by truncation of the PG domain, surface expression of CAIX was determined by antibody staining of fixed and permeabilized oocytes, using an antibody mapping against the intracytoplasmic (IC) region of CAIX (Figure 2A-2D). Oocytes coexpressing MCT1/4 with either CAIX-WT or CAIX-ΔPG showed robust signals for CAIX at the cell surface, indicating that both CAIX-WT and CAIX-ΔPG are integrated equally well into the plasma membrane of *Xenopus* oocytes. Native oocytes displayed only very weak staining for CAIX, showing specificity of the antibody (Figure 2E).

### CAIX-mediated facilitation of lactate transport can be inhibited by an antibody against the PG domain

**Figure 3:**
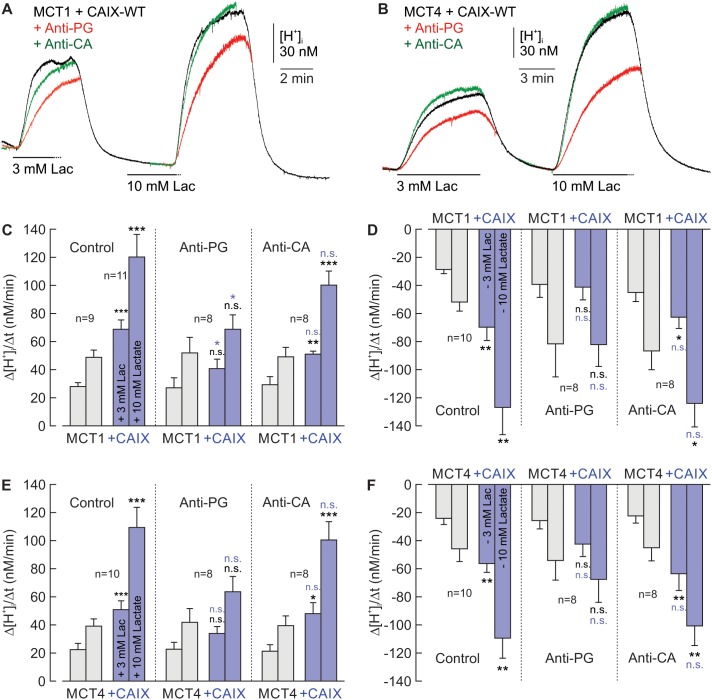
Antibodies directed against the PG domain, but not against the catalytic domain of CAIX impair functional interaction between MCT1/4 and CAIX **(A, B)** Original recordings of the change in intracellular H^+^ concentration ([H^+^]_i_) in oocytes coexpressing MCT1+CAIX-WT (A) and MCT4+CAIX (B), respectively, during application of 3 and 10 mM lactate. Cells were pre-incubated for 24 h with 0.4 μg/ml Anti-PG (red traces), 0.4 μg/ml Anti-CA (green traces) or without antibodies (black traces) before the measurements were carried out. **(C, D)** Rate of change in intracellular H^+^ concentration (Δ[H^+^]/Δt) as induced by application **(C, E)** and removal **(D, F)** of 3 and 10 mM lactate, in oocytes expressing MCT1/4 (gray) and MCT1/4+CAIX-WT (blue), respectively, in the presence of Anti-PG, Anti-CA or in the absence of antibodies (control). Data are represented as mean + SEM. Significance in differences was tested with ANOVA, followed by means comparison. The black significance indicators above the bars for MCT1/4+CAIX-coexpressing oocytes refer to the corresponding values from oocytes expressing MCT1/4 alone (gray bars). The blue significance indicators above the bars for MCT1/4+CAIX-coexpressing oocytes, incubated with Anti-PG or Anti-CA refer to the values from MCT1/4+CAIX-coexpressing oocytes not incubated with antibody (blue bars). No significant changes were found between MCT1/4-expressing oocytes incubated with or without antibodies (gray bars).

Since CAIX requires its PG domain to facilitate MCT transport activity, we investigated whether an antibody, directed against the PG domain (Anti-PG; also termed M75 [16]), could inhibit the CAIX-mediated increase in lactate transport. Therefore we expressed MCT1 and MCT4 in *Xenopus* oocytes, either alone or together with CAIX-WT. Oocytes were incubated for 24h in a solution containing either 0.4 μg/ml Anti-PG, or 0.4 μg/ml of an antibody directed against the catalytic domain (Anti-CA; also termed V/10 [23]), or no antibody at all. Transport activity of MCT1 and MCT4 was again determined by measuring Δ[H^+^]_i_/Δt during application and withdrawal of lactate (Figure 3A, 3B). Indeed Anti-PG inhibited the CAIX-induced increase in transport activity of MCT1 (Figure 3C, 3D) and MCT4 (Figure 3E, 3F), as indicated by the significant reduction in Δ[H^+^]_i_/Δt in MCT1/4+CAIX-expressing oocytes in the presence of Anti-PG. In oocytes expressing MCT1 and MCT4 without CAIX no change in Δ[H^+^]_i_/Δt was observed in the presence of Anti-PG, indicating that Anti-PG had no effect on MCT1 and MCT4 transport function in the absence of CAIX (Figure 3C-3F, gray bars). In contrast to Anti-PG, Anti-CA, directed against the catalytic domain of CAIX, was not able to reduce the CAIX-mediated augmentation of MCT transport activity (Figure 3C-3F). This result is in line with our previous finding that inhibition of CAIX catalytic activity with the CA inhibitor 6-Ethoxy-2-benzothiazolesulfonamide (EZA) does not inhibit CAIX-driven lactate transport in oocytes and cancer cells [18].

**Figure 4:**
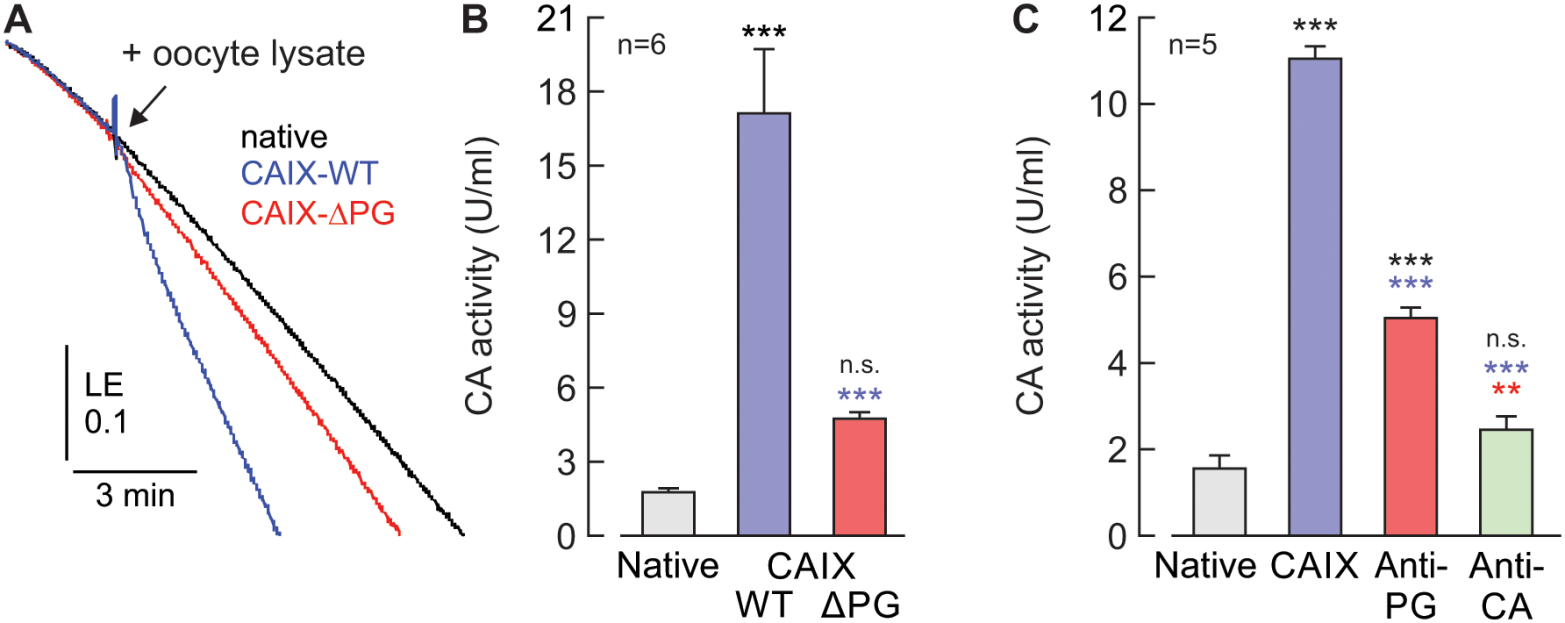
The PG domain of CAIX facilitates catalytic activity *in vitro* **(A)** Original recordings of the log enrichment (LE), as measured by gas-analysis mass spectrometry, of a pool of 20 lysed, native oocytes (black trace) and pools of 20 lysed oocytes, expressing either CAIX-WT (blue trace) or CAIX-ΔPG (red trace). The beginning of the traces shows the rate of degradation of the ^18^O-labeled substrate in the non-catalyzed reaction. The black arrowhead indicates addition of oocyte lysate. **(B)** Enzymatic activity of lysates from 20 native oocytes (gray) and 20 oocytes expressing either CAIX-WT (blue) or CAIX-ΔPG (red), respectively. One unit is defined as 100% stimulation of the non-catalyzed ^18^O depletion of doubly labelled ^13^C^18^O_2_. Data are represented as mean + SEM. Significance in differences was tested with ANOVA, followed by means comparison. The black significance indicators above the bars from CAIX-expressing oocytes refer to the values from native cells (gray bar). The blue asterisks above the bar from CAIX-ΔPG-expressing oocytes refer to the values from CAIX-WT-expressing oocytes (blue bar). **(C)** Enzymatic activity of cell lysate from 20 native (gray) and CAIX-expressing oocytes (blue, red and green), respectively. Cell lysates were incubated for 2h with 5 μg/ml of Anti-PG (red), 5 μg/ml of Anti-CA (green), or without antibody (blue). One unit is defined as 100% stimulation of the non-catalyzed ^18^O depletion of doubly labelled ^13^C^18^O_2_. Data are represented as mean + SEM. Significance in differences was tested with ANOVA, followed by means comparison. The black significance indicators above the bars from CAIX-expressing oocytes refer to the values from native cells (gray bar). The blue asterisks above the bar from CAIX-expressing oocytes, incubated with antibodies refer to the values from CAIX-WT-expressing oocytes without antibody (blue bar). The red asterisks above the bar from CAIX-expressing oocytes, incubated with Anti-CA refer to the values from CAIX-WT-expressing oocytes, incubated with Anti-PG (red bar).

Among other functions, the PG domain has been suggested to enhance CAIX catalytic activity [15]. Determination of CA catalytic activity in oocyte lysates by gas analysis mass spectrometry indicated that truncation of the CAIX-PG domain leads to a significant reduction in catalytic activity, as compared to CAIX-WT (Figure 4A, 4B). In line with this, incubation of lysates from CAIX-WT-expressing oocytes with 5 μg/ml of Anti-PG also resulted in a significant decrease of CA catalytic activity, as did incubation of oocyte lysates with 5 μg/ml of Anti-CA (Figure 4C).

### Hypoxia-induced augmentation of lactate transport in breast cancer cells can be inhibited by an antibody against the CAIX-PG domain

**Figure 5:**
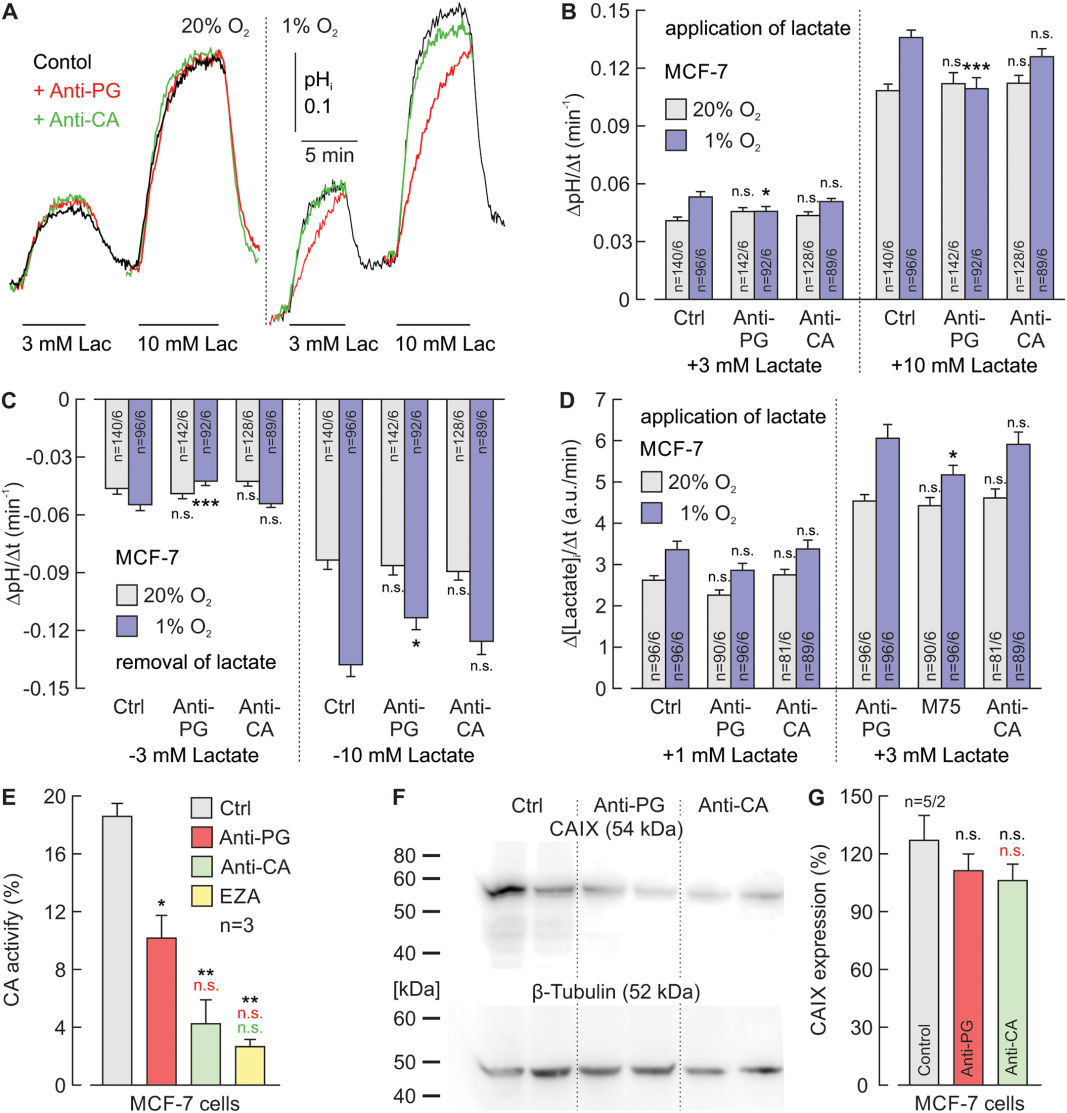
Antibodies directed against the PG domain, but not against the catalytic domain of CAIX reduce lactate transport in hypoxic MCF-7 cells **(A)** Original recordings of the change in intracellular pH (pH_i_) during application of 3 and 10 mM of lactate in MCF-7 breast cancer cells, incubated under normoxia (left traces) or hypoxia (right traces), in the presence of 5 μg/ml Anti-PG (red traces), 5 μg/ml Anti-CA (green traces) or in the absence of antibodies (black traces). **(B, C)** Rate of change in intracellular pH, as induced by application (B) and removal (C) of lactate in MCF-7 cells, incubated under normoxia (gray) or hypoxia (blue), in the presence of Anti-PG, Anti-CA or in the absence of antibodies (Ctrl). **(D)** Relative change in intracellular lactate concentration, as induced by application of lactate in MCF-7 cells, incubated under normoxia (gray) or hypoxia (blue) in the presence of Anti-PG, Anti-CA or in the absence of antibodies (Ctrl). Data are represented as mean ± SEM. Significance in differences was tested with ANOVA, followed by means comparison. The significance indicators above the bars from cells, incubated with antibody refer to the corresponding values from control cells (Ctrl). **(E)** CA catalytic activity of MCF-7 cells, as measured by gas-analysis mass spectrometry, in the presence 5 μg/ml Anti-PG (red), 5 μg/ml Anti-CA (green), 30 μM of the CA inhibitor EZA (yellow) and in the absence of antibodies or inhibitors (Ctrl). Data are represented as mean + SEM. Significance in differences was tested with ANOVA, followed by means comparison. The black asterisks above the bars from cells treated with antibodies or EZA refer to the values from control cells (gray bar). The red asterisks above the bars from cells treated with Anti-CA or EZA refer to the values from cells treated with Anti-PG (red bar). The green significance indicator above the bar from cells treated with EZA refers to the values from cells treated with Anti-CA (green bar). **(F)** Western blot against CAIX and β-tubulin as loading control, in MCF-7 cells, incubated in the presence Anti-PG, Anti-CA or in the absence of antibodies (Ctrl). **(G)** Quantification of the protein level of CAIX in MCF-7 cells, incubated in the presence Anti-PG (red), Anti-CA (green) or in the absence of antibodies (gray) relative to the protein level of β-tubulin. Data are represented as mean + SEM. Significance in differences was tested with ANOVA, followed by means comparison. The black significance indicators above the bars from cells, incubated with antibody refer to the corresponding values from control cells (gray bar). The red significance indicator above the bar from cells treated with Anti-CA refers to the values from cells treated with Anti-PG (red bar).

**Figure 6:**
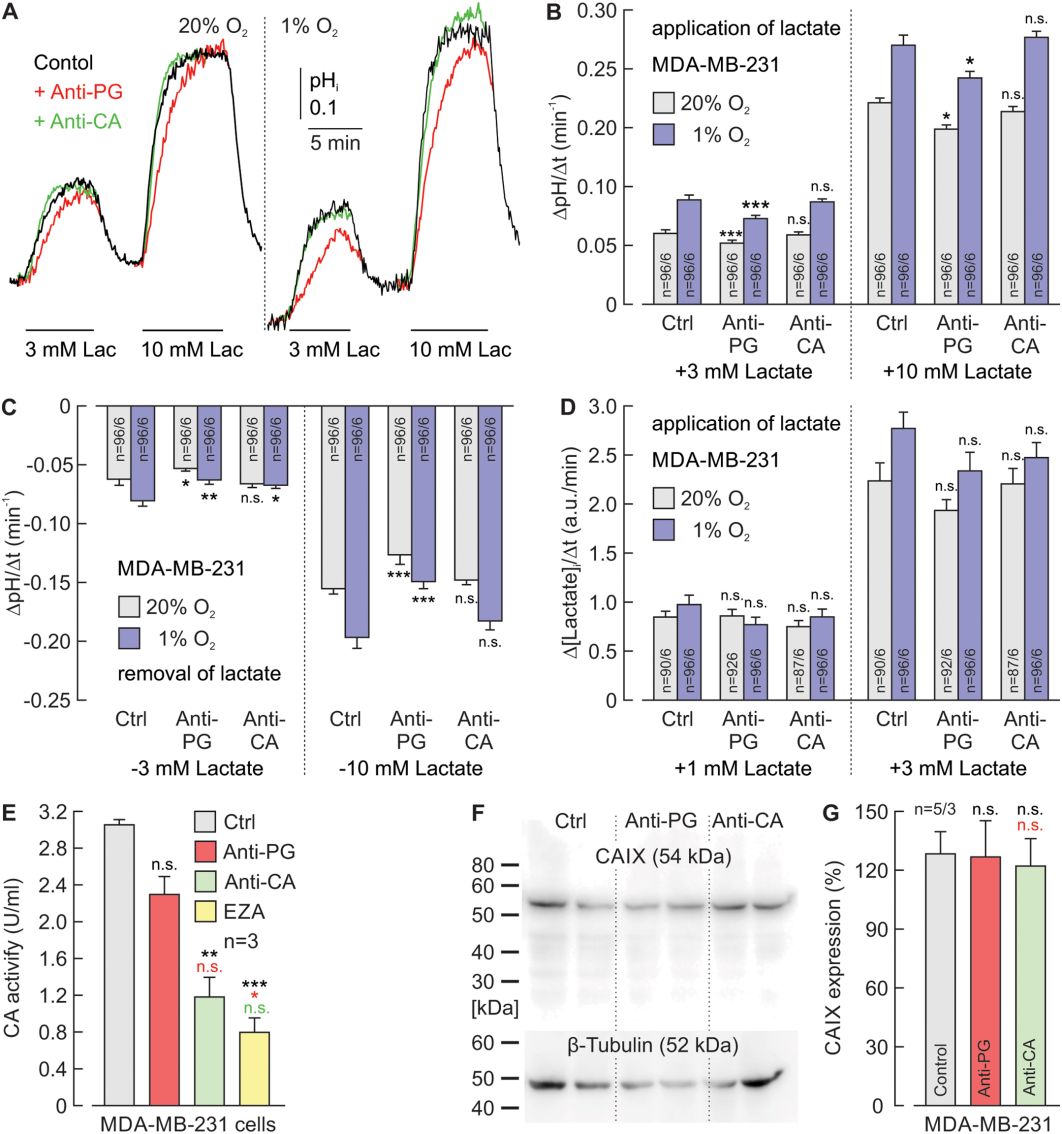
Antibodies directed against the PG domain, but not against the catalytic domain of CAIX reduce lactate transport in MDA-MB-231 cells **(A)** Original recordings of the change in intracellular pH (pH_i_) during application of 3 and 10 mM of lactate, in MDA-MB-231 breast cancer cells, incubated under normoxia (left traces) or hypoxia (right traces), in the presence of 5 μg/ml Anti-PG (red traces), 5 μg/ml Anti-CA (green traces) or in the absence of antibodies (black traces). **(B, C)** Rate of change in intracellular pH, as induced by application (B) and removal (C) of lactate in MDA-MB-231 cells, incubated under normoxia (gray) or hypoxia (blue), in the presence of Anti-PG, Anti-CA or in the absence of antibodies (Ctrl). **(D)** Relative change in intracellular lactate concentration, as induced by application of lactate in MDA-MB-231 cells, incubated under normoxia (gray) or hypoxia (blue) in the presence of Anti-PG, Anti-CA or in the absence of antibodies (Ctrl). Data are represented as mean ± SEM. Significance in differences was tested with ANOVA, followed by means comparison. The significance indicators above the bars from cells, incubated with antibody refer to the corresponding values from control cells (Ctrl). **(E)** CA catalytic activity of MDA-MB-231 cells, as measured by gas-analysis mass spectrometry, in the presence 5 μg/ml Anti-PG (red), 5 μg/ml Anti-CA (green), 30 μM of the CA inhibitor EZA (yellow) and in the absence of antibodies or inhibitors (Ctrl). Data are represented as mean + SEM. Significance in differences was tested with ANOVA, followed by means comparison. The black asterisks above the bars from cells treated with antibodies or EZA refer to the values from control cells (gray bar). The red asterisks above the bars from cells treated with Anti-CA or EZA refer to the values from cells treated with Anti-PG (red bar). The green significance indicator above the bar from cells treated with EZA refers to the values from cells treated with Anti-CA (green bar). **(F)** Western blot against CAIX and β-tubulin as loading control, in MDA-MB-231 cells, incubated in the presence Anti-PG, Anti-CA or in the absence of antibodies (Ctrl). **(G)** Quantification of the protein level of CAIX in MDA-MB-231 cells, incubated in the presence Anti-PG (red), Anti-CA (green) or in the absence of antibodies (gray) relative to the protein level of β-tubulin. Data are represented as mean + SEM. Significance in differences was tested with ANOVA, followed by means comparison. The black significance indicators above the bars from cells, incubated with antibody refer to the corresponding values from control cells (gray bar). The red significance indicator above the bar from cells treated with Anti-CA refers to the values from cells treated with Anti-PG (red bar).

To investigate whether interference with the CAIX PG domain can also inhibit the hypoxia-induced augmentation of proton-coupled lactate transport in cancer cells, we incubated MCF-7 (Figure 5) and MDA-MB-231 (Figure 6) breast cancer cells under normoxia (20% O_2_) or hypoxia (1% O_2_) in the presence of 5 μg/ml Anti-PG, 5 μg/ml Anti-CA, or without antibodies (Ctrl). MCT transport activity was determined by measuring the rate of change in pH_i_ (ΔpH_i_/Δt) during application and removal of lactate, using the pH-sensitive fluorescent dye BCECF (Figure 5A-5C; Figure 6A-6C) and by measuring the relative rate of change in intracellular lactate concentration (Δ[Lactate]_i_/Δt) during lactate application with the lactate-sensitive FRET nano-sensor *Laconic* (Figure 5D; Figure 6D). In the absence of antibodies, both proton-coupled lactate influx, as measured by the rate of change in pH_i_ and [lactate]_i_, during application of lactate and proton-coupled lactate efflux, as measured by the rate of change in pH_i_ during removal of lactate were slightly but significantly increased in hypoxic MCF-7 and MDA-MB-231 cells (Figure 5A-5D; Figure 6A-6D). This hypoxia-induced increase in transport activity was significantly reduced by Anti-PG in both cell lines, while incubation with Anti-CA led to no significant changes in transport activity. These results indicate that Anti-PG, which is directed against the CAIX-PG domain, can inhibit the functional interaction between MCTs and CAIX in cancer cells.

The influence of Anti-PG and Anti-CA on CAIX catalytic activity in MCF-7 and MDA-MB-231 cells was investigated by gas analysis mass spectrometry (Figure 5E; Figure 6E). Anti-PG decreased CA catalytic activity in hypoxic MCF-7 and MDA-MB-231 cells to 55% and 75%, respectively. In the presence of Anti-CA, CA activity decreased to 23% and 39%, respectively, values that did not significantly differ from the catalytic activity observed in the presence of EZA.

To make sure that the observed effects on lactate transport and catalytic activity are not due to a decrease in the CAIX protein level during incubation with the antibodies, CAIX protein levels were determined in hypoxic MCF-7 and MDA-MB-231 cells by western blot analysis (Figure 5F; Figure 6F). Quantification of the blots demonstrated that neither Anti-PG nor Anti-CA induced a significant decrease in CAIX protein levels in MCF-7 and MDA-MB-231 cells (Figure 5G; Figure 6G).

### CAIX mediates facilitation of MCT transport activity independent of the lactate concentration

**Figure 7:**
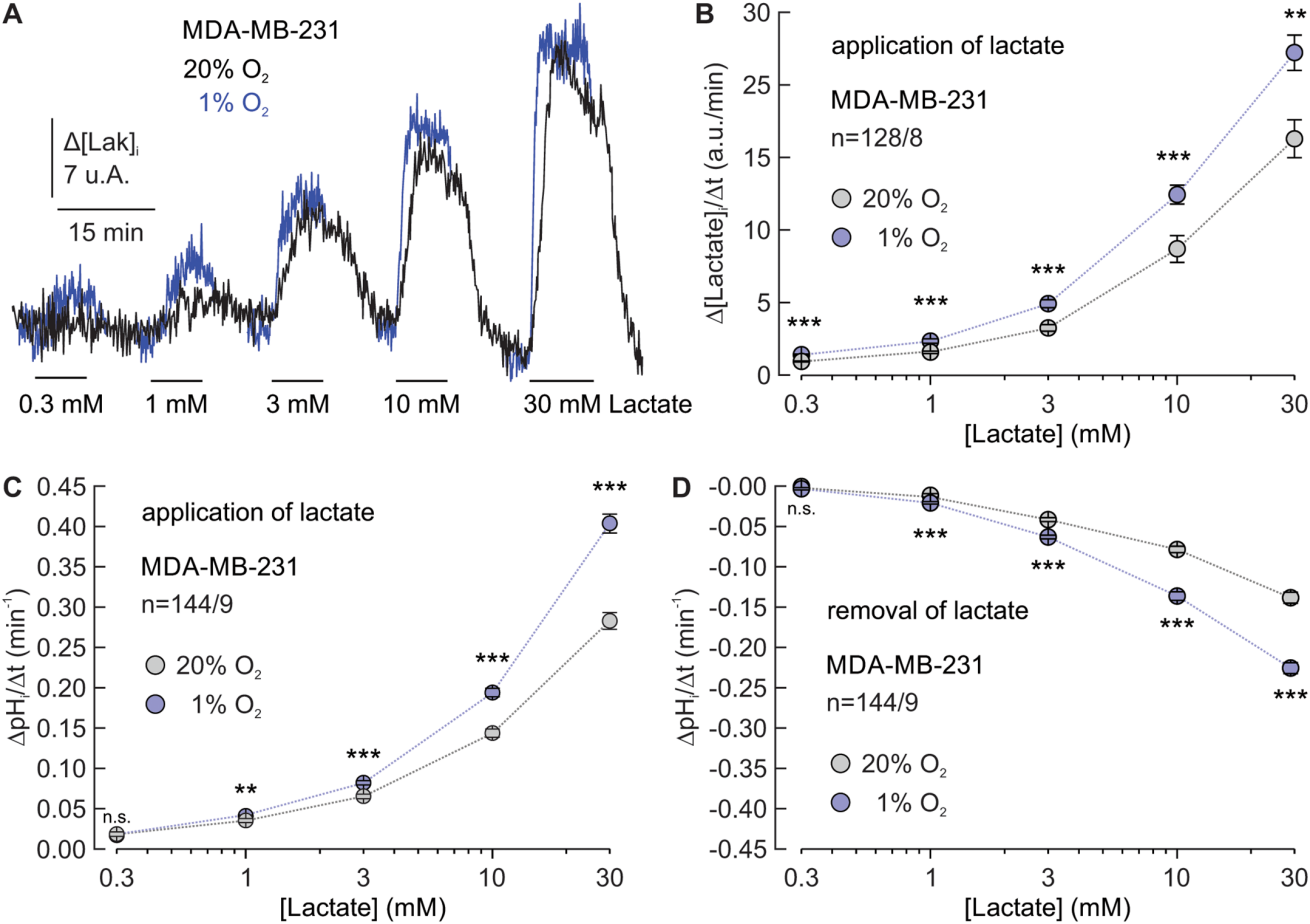
CAIX mediates facilitation of MCT transport activity independent of the lactate concentration **(A)** Original recordings of the relative change in intracellular lactate concentration during application of 0.3, 1, 3, 10, and 30 mM of lactate, in MDA-MB-231 breast cancer cells, incubated under normoxia (black traces) or hypoxia (blue traces). **(B)** Relative change in intracellular lactate concentration, as induced by application of lactate in MDA-MB-231 cells, incubated under normoxia (gray) or hypoxia (blue), plotted against the extracellular lactate concentration. **(C, D)** Rate of change in intracellular pH, as induced by application (C) and removal (D) of lactate in MDA-MB-231 cells, incubated under normoxia (gray) or hypoxia (blue), plotted against the extracellular lactate concentration. Data are represented as mean ± SEM. Significance in differences was tested with Student's t-test, following a Shapiro-Wilk test. The significance indicators above each pair of dots refer to the difference between normoxic and hypoxic cells at the respective lactate concentration.

Cancer cells show a high variability in glycolytic energy metabolism, resulting in an equivalently high variability in the rate of cellular lactate production. To investigate whether the hypoxia-induced facilitation of proton-coupled lactate transport can also take place over such a brought range of lactate concentrations, we measured proton-coupled lactate transport in normoxic and hypoxic MDA-MB-231 cells during application and removal of lactate at different concentrations, ranging from 0.3 to 30 mM (Figure 7A-7D). MCT transport activity was again determined by measuring the relative rate of change in intracellular lactate concentration (Δ[Lactate]_i_/Δt) during lactate application with the lactate-sensitive FRET nano-sensor *Laconic* (Figure 7A, 7B) and by measuring ΔpH_i_/Δt during application and removal of lactate, using the pH-sensitive fluorescent dye BCECF (Figure 7C, 7D). Lactate influx significantly increased by 40% to 50% in hypoxic cells at all lactate concentrations (Figure 7A, 7B). Proton influx and efflux were also significantly increased under hypoxia at all lactate concentrations, with the exception of the lowest concentration of 0.3 mM (Figure 7C, 7D). Application and removal of 0.3 mM lactate leads to only very small changes in pH_i_, which are sometimes even indistinguishable from the background noise. Therefore it is likely that a hypoxia-induced increase in ΔpH_i_/Δt during application of 0.3 mM lactate is masked by noise in the measurements. There was no clear tendency for a stronger facilitation of proton-coupled lactate transport at higher or lower lactate concentrations. From these results it can be concluded that CAIX facilitates lactate transport in hypoxic cancer cells over a brought range of lactate concentrations, to allow efficient lactate efflux already at low intracellular lactate levels, but also when the cell produces lactate at the highest rates.

### Anti-PG inhibits cell proliferation and migration of breast cancer cells

**Figure 8:**
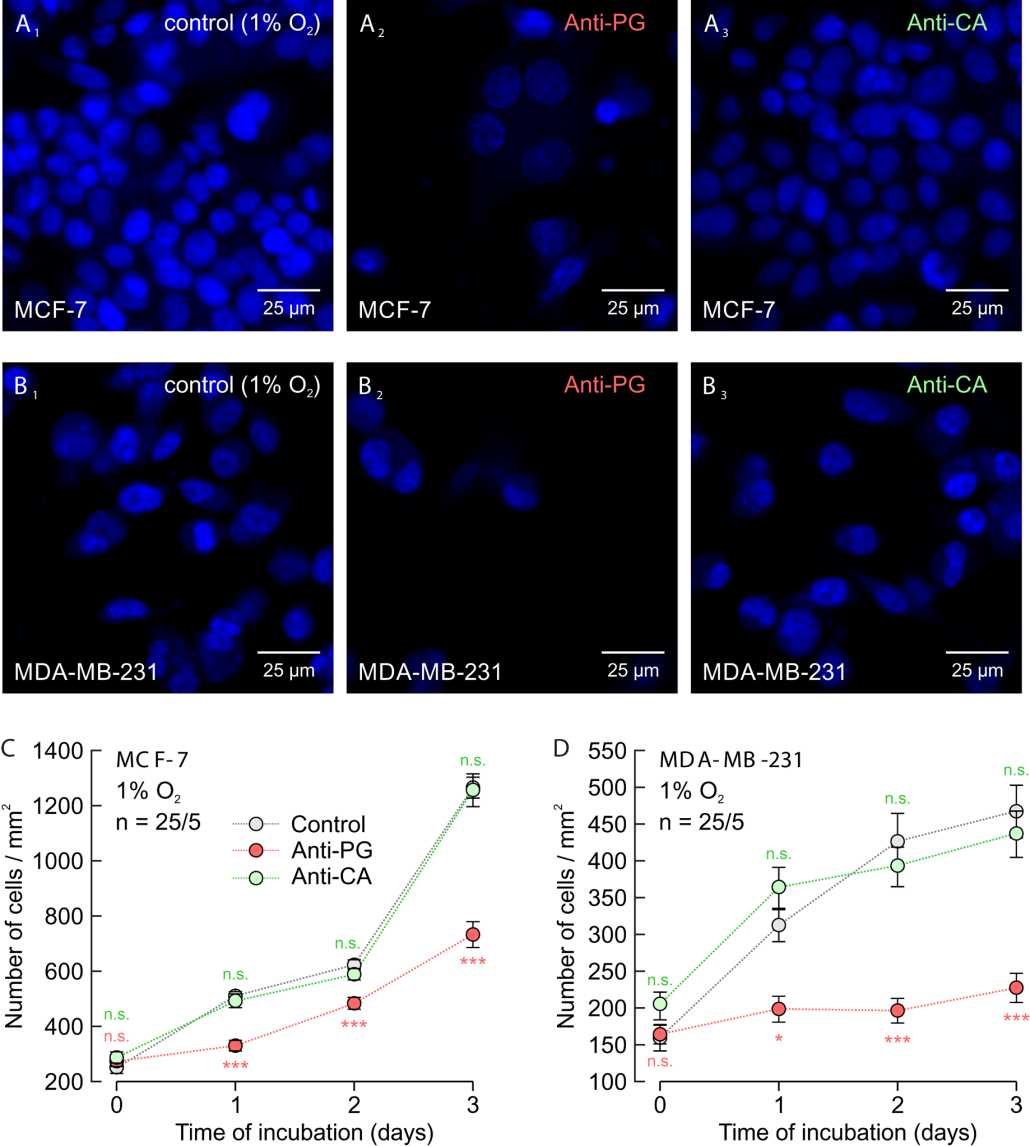
Antibodies directed against the PG domain, but not against the catalytic domain of CAIX inhibit proliferation of MCF-7 and MDA-MB-231 cells **(A, B)** Staining of nuclei with Hoechst (blue) in MCF-7 (A) and MDA-MB-231 cells (B) after 3 days in culture at 1% O_2_. Hypoxic cells remained either untreated (A_1_, B_1_) or were incubated with 5 μg/ml Anti-PG (A_2_, B_2_) or 5 μg/ml Anti-CA (A_3_, B_3_). **(C, D)** Total number of nuclei/mm^2^ in MCF-7 (C) and MDA-MB-231 (D) cell cultures, kept for 0–3 days under the conditions as described in (A and B). Data are represented as mean ± SEM. Significance in differences was tested with ANOVA, followed by means comparison. The red significance indicators depict differences between cells treated with Anti-PG and control cells, the green significance indicators depict differences between cells treated with Anti-CA and control cells at the respective time points.

To test whether application of Anti-PG, which inhibits the hypoxia-induced increase in lactate transport, could also inhibit proliferation of cancer cells, we measured cell proliferation in hypoxic MCF-7 and MDA-MB-231 cells for up to three days (Figure 8A-8D). Indeed incubation of cells with 5 μg/ml of Anti-PG led to a significant decrease in cell proliferation, both in MCF-7 and MDA-MB-231 cells, while incubation with 5 μg/ml of Anti-CA resulted in no significant effects.

**Figure 9:**
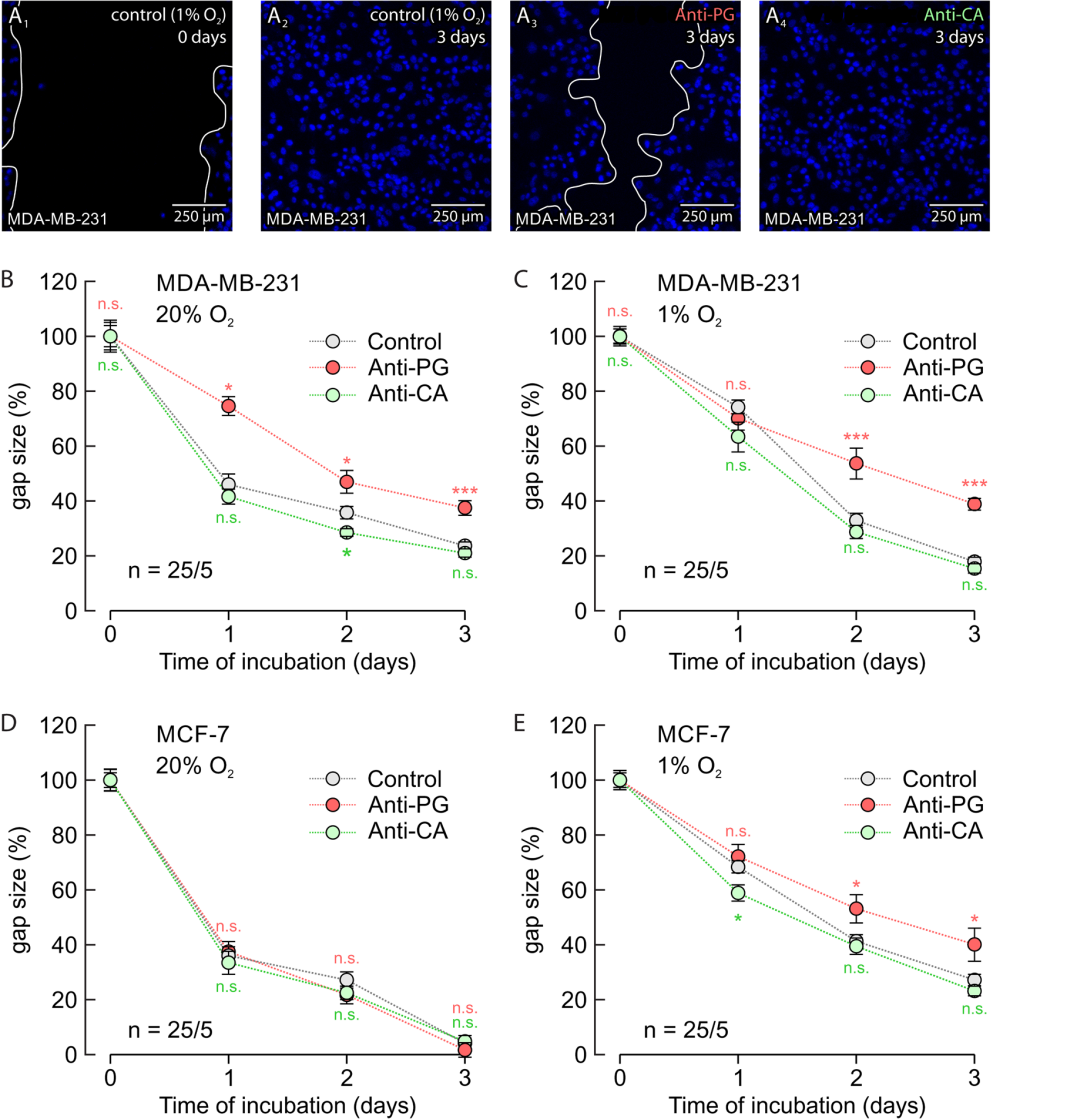
Antibodies directed against the PG domain, but not against the catalytic domain of CAIX inhibit migration of MDA-MB-231 and MCF-7 cells **(A)** Staining of nuclei with Hoechst (blue) in hypoxic MDA-MB-231 cells before (A_1_) and 3 days after production of a scratch through the culture (A_2_-A_4_). Cells remained either untreated (A_1_, A_2_) or where incubated with Anti-PG (A_3_) or Anti-CA (A_4_). **(B-E)** Size of the gap (%) in MDA-MB-231 (B, C) and MCF-7 (D, E) cell cultures, 0–3 days after scratching. Cells were incubated under normoxic (B, D) or hypoxic (C, E) conditions in the presence of Anti-PG (red) and Anti-CA (green), respectively, or without antibody (gray). Data are represented as mean ± SEM. Significance in differences was tested with ANOVA, followed by means comparison. The red significance indicators depict differences between cells treated with Anti-PG and control cells, the green significance indicators depict differences between cells treated with Anti-CA and control cells at the respective time points.

**Figure 10:**
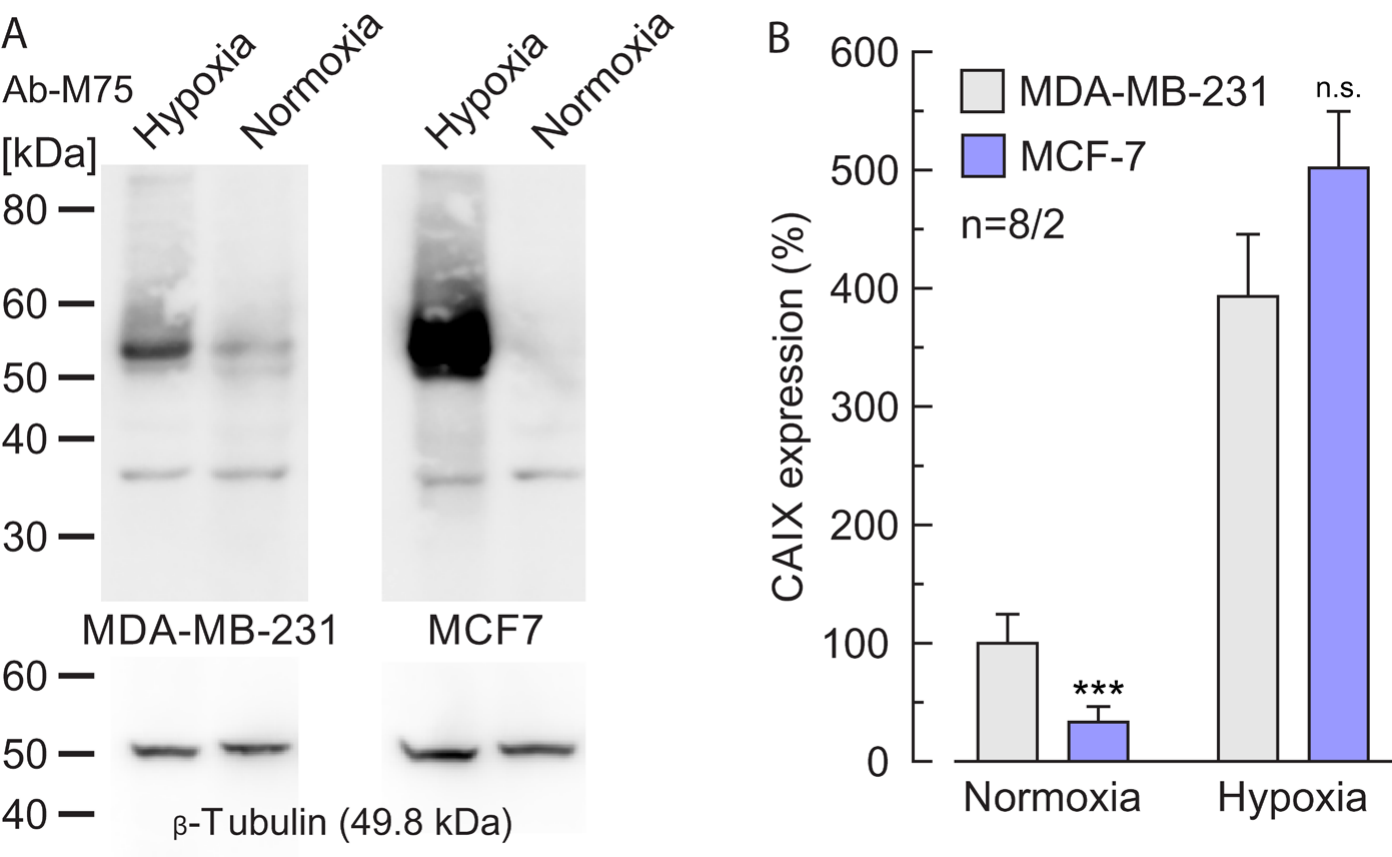
Expression levels of CAIX in normoxic and hypoxic MCF-7 and MDA-MB-231 cells **(A)** Western blot of CAIX, in normoxic and hypoxic MCF-7 and MDA-MB-231 cells with Anti-PG (Ab-M75, upper blots). β-tubulin was used as loading control (lower blots). **(B)** Quantification of the protein level of CAIX in normoxic and hypoxic MDA-MB-231 and MCF-7 cells, respectively, relative to the protein level of β-tubulin. Data are represented as mean + SEM. Significance in differences was tested with Student's t-test, following a Shapiro-Wilk test. The significance indicators above the bars for normoxic and hypoxic MCF-7 cells refer to the corresponding values for normoxic and hypoxic MDA-MB-231 cells (gray bars).

To investigate the influence of Anti-PG and Anti-CA on cell migration we performed a scratch assay on normoxic and hypoxic MCF-7 and MDA-MB-231 cells (Figure 9A-9E). Under hypoxic conditions 5 μg/ml Anti-PG inhibited migration of both MCF-7 and MDA-MB-231 cells (Figure 9C, 9E). Under normoxic conditions, however, 5 μg/ml Anti-PG only inhibited migration of MDA-MB-231 cells (Figure 9B), but had no effect on migration of MCF-7 cells (Figure 9D). This difference can be attributed to differences in the CAIX expression level in MCF-7 and MDA-MB-231 cells. Western blots showed that MDA-MB-231 cells already show a robust expression level of CAIX under normoxia, while MCF-7 cells only express very low amounts of CAIX (Figure 10A, 10B; Ref. [18]). This difference in the CAIX expression level could explain why application of Anti-PG under normoxia does only have an effect on cell migration in MDA-MB-231 but not in MCF-7 cells. 5 μg/ml of Anti-CA showed no effect on cell migration under any condition (Figure 9B-9D).

## DISCUSSION

We have previously reported that CAIX facilitates transport activity of MCTs in hypoxic MCF-7 breast cancer cells and *Xenopus* oocytes by a mechanism which is independent of the enzyme's catalytic activity [18]. In the present study, we demonstrate that CAIX-mediated facilitation of MCT transport activity in both *Xenopus* oocytes and cancer cells requires the CAIX proteoglycan-like domain, which is rich in negatively charged amino acids. If several negatively charged groups are located near each other at the protein surface, the proton collective properties of this surface may increase the protonation rate of a specific group, like the proton-transfer pathway of a membrane protein, a phenomenon termed ‘proton antenna’ [19]. The PG domain of human CAIX contains 18 glutamate and 8 aspartate residues, which have already been suggested to function as a proton buffer [15]. Since these 26 negatively charged side chains are located very close to each other in the peptide chain (Figure 1A), it appears plausible that (some of) these protonatable residues could function as a ‘proton antenna’ for the transporter. This assumption is further strengthened by the finding that CAIX-mediated facilitation of H^+^/lactate cotransport was also decreased by application of an antibody, directed against the PG domain (Anti-PG), but not by an antibody, directed against the enzyme's catalytic domain (Anti-CA). These data are in line with our previous findings that CAIX-mediated facilitation of lactate flux in cancer cells and *Xenopus* oocytes does not require CAIX catalytic activity, but seems to be mediated by CAIX, functioning as a ‘proton antenna’ for the transporter [18].

Besides reducing the CAIX-mediated increase in H^+^/lactate cotransport, Anti-PG also decreased proliferation and migration of MCF-7 and MDA-MB-231 breast cancer cells, while Anti-CA had no effect on these two parameters. We could recently show that knockdown of CAIX with siRNA, but not inhibition of CAIX catalytic activity with EZA decreases proliferation of hypoxic MCF-7 and MDA-MB-231 cells [18], suggesting that non-catalytic facilitation of MCT-mediated lactate efflux via CAIX can support cell proliferation under hypoxic conditions. The observation that Anti-PG inhibits cell proliferation, like knockdown of CAIX, supports the assumption that the CAIX PG domain can facilitate lactate transport across the cell membrane to support tumor cell proliferation. Furthermore, a multitude of studies has demonstrated that CAIX can promote cell migration by direct interaction with the cytoskeleton and by influencing intracellular and extracellular H^+^ concentrations. On the one hand CAIX interacts with β-catenin and thus interferes with the E-cadherin attachment to the actin cytoskeleton. This interaction destabilizes cell-cell adhesions and thus significantly supports cell migration [24, 25]. Moreover, the PG domain of CAIX facilitates cell adhesion and spreading on solid support and participates in formation of focal contacts, which are involved in both cell migration (particularly in transient attachment of protruding lamellipodia) and in proliferation (through activation of FAK-regulated mitogenic and metabolic pathways) [17, 26]. On the other hand, the CAIX was shown to support the formation of intracellular and pericellular pH gradients from the leading edge to the trailing edge of a migrating cell, which is one of the prerequisites for cell migration [27]. This pH gradient is created by the concerted activity of various acid/base transporters, including the Na^+^/H^+^ exchanger NHE1, the Na^+^/HCO_3_^-^ cotransporter NBC, the Cl^-^/HCO_3_^-^ exchanger AE2, and MCT4, which relocate from their original position into the leading edge [28]. In this membrane region CAIX has been shown to colocalize with NBCe1 and AE2, suggesting that CAIX could form a transport metabolon with these carriers [29]. Up to now no direct interaction between MCTs and CAIX in the leading edge of migrating cells has been shown. However, it has been demonstrated that knockdown of MCT4 slows migration in oral squamous cell carcinoma and retinal pigment epithelium cell lines [30, 31]. These studies suggest that extrusion of lactate and protons via MCTs can support cell migration. Since interference with the CAIX PG domain by Anti-PG reduces MCT transport capacity, it appears plausible that the reduction in cell migration, as observed in the presence of Anti-PG can, at least to some extent, be attributed to reduced H^+^/lactate efflux via MCT1 and MCT4. Nevertheless, the observed reduction in migration is most likely the result of various effects of CAIX including its interaction with MCTs, NHE1, NBC, AE2, and the cytoskeleton. Therefore further experiments are required to fully understand the role of the CAIX PG domain in cancer cell migration.

It has been suggested previously that the acidic residues in the PG domain could function as a proton buffer to facilitate CAIX catalytic activity [15]. Indeed, we could show that both truncation of the PG domain and application of Anti-PG resulted in a significant decrease in CAIX catalytic activity when measured by gas analysis mass spectrometry. However, when CAIX activity was determined by measuring the rate of rise in intracellular H^+^ concentration during application of CO_2_/HCO_3_^-^ in intact oocytes, no difference could be observed between CAIX-WT and CAIX-ΔPG. The discrepancy between these two results could be explained by the differences in the localization of the carbonic anhydrases measured. MS measurements on oocyte lysates or cell suspensions determine CA catalytic activity in HEPES-buffered salt solutions that mimic the extracellular space. On the other side, the rate of rise in intracellular H^+^ concentration during application of CO_2_/HCO_3_^-^ in intact oocytes measures CA activity inside the cell's cytosol. Indeed, we have recently shown that ca. 80% of CAIX is found in the cytosol, when the enzyme is heterologously expressed in *Xenopus* oocytes [32]. Therefore intracellular pH measurements primarily determine intracellular CA activity, while MS measurement in cell lysates and suspensions primarily determine extracellular activity. Therefore it can be assumed that the proton buffer of the PG domain is only required to facilitate catalytic activity of extracellular CAIX, but not when CAIX is localized in the cytosol, which contains high amounts of mobile buffers which could substitute for the additional proton buffers in the PG domain.

Taken together the results indicate that the PG domain of CAIX does not only provide a proton buffer for the enzyme to enhance catalytic activity, but could also serve as ‘proton antenna’ for monocarboxylate transporters to facilitate proton-driven lactate transport in hypoxic cancer cells, thereby supporting tumor cell proliferation and migration.

## MATERIALS AND METHODS

### Antibodies

Production and characterization of the antibodies M75 (directed against the CAIX PG domain; termed Anti-PG in the present study) and V/10 (directed against the CAIX catalytic domain (CA); termed Anti-CA in the present study) has been described in detail previously [17, 23, 33]. In brief, antibodies were isolated from mouse hybridoma cell lines, immunized with GST-fusion proteins of the CAIX PG domain and the CAIX CA domain, respectively. Characterization of M75 and V/10 was carried out by ELISA and immunohistochemistry [23]. The results from this study suggest that M75 and V/10 exhibit similar affinities to CAIX, when cells are incubated over a longer time period with the antibodies. In the present study culture medium from hybridoma cells, containing either 100 μg/ml of M75 or 100 μg/ml of V/10 was used. Incubation of intact oocytes (which are kept in a saline without serum) was carried out with 0.4 μg/ml of M75 or V/10. Cells were incubated for 24 hours to ensure maximum binding of the antibodies. For incubation of oocyte lysates, the antibody concentration was increased to 5 μg/ml of M75 or V/10, to account for unspecific binding of the antibodies to intracellular substances, while the incubation time was decreased to two hours to reduce degradation of CAIX protein in the cell lysate. Incubation of MCF-7 and MDA-MB-231 cell lines was carried out with 5 μg/ml of M75 or V/10, to account for unspecific binding of the antibodies to compounds in the serum, added to the culture medium. Cells were incubated for 24 hours to ensure maximum binding of the antibodies.

### Heterologous protein expression in *Xenopus* oocytes

cDNA coding for human CAIX-WT, a CAIX mutant with truncated PG domain (CAIX-ΔPG), rat MCT1, and rat MCT4, all cloned into the oocyte expression vector pGEM-He-Juel, were transcribed *in vitro* with T7 RNA-Polymerase (mMessage mMachine, Ambion Inc., Austin, USA) as described earlier [33, 34]. *Xenopus laevis* females were purchased from the Radboud University, Nijmegen, Netherlands. Segments of ovarian lobules were surgically removed under sterile conditions from frogs anaesthetized with 3 g/l of ethyl 3-aminobenzoate methanesulfonate (MS-222, Sigma-Aldrich), and rendered hypothermic. The procedure was approved by the Landesuntersuchungsamt Rheinland-Pfalz, Koblenz (23 177-07/A07-2-003 §6) and the Niedersächsisches Landesamt für Verbraucherschutz und Lebensmittelsicherheit, Oldenburg (33.19-42502-05-17A113). As described earlier [34, 35], oocytes were singularized by collagenase (Collagenase A, Roche, Mannheim, Germany) treatment in Ca^2+^-free oocyte saline (pH 7.8) at 28°C for up to 2 h. The singularized oocytes were left overnight in an incubator at 18°C in Ca^2+^-containing oocyte saline (pH 7.8) to recover. Oocytes of the stages V and VI were injected with 5 ng of cRNA coding for MCT1 or MCT4, either together with 5 ng of cRNA coding for CAIX, CAIX-ΔPG, or alone. Measurements were carried out 3 to 6 days after injection of cRNA. The oocyte saline had the following composition: 82.5 mM NaCl, 2.5 mM KCl, 1 mM CaCl_2_, 1 mM MgCl_2_, 1 mM Na_2_HPO_4_, 5 mM HEPES; titrated with NaOH to the desired pH. In CO_2_/HCO_3_^-^- and lactate-containing saline, NaCl was substituted by NaHCO_3_ or Na-L-lactate in equimolar amounts.

### Measurement of intracellular H^+^ concentration in *Xenopus* oocytes

Changes in intracellular H^+^ concentration in oocytes were determined with ion-sensitive microelectrodes under voltage-clamp conditions, using single-barreled microelectrodes; the manufacture and application have been described in detail previously [35, 36]. Briefly, a borosilicate glass capillary of 1.5 mm in diameter was pulled to a micropipette and was silanized with a drop of 5% tri-N-butylchlorsilane in 99.9% pure carbon tetrachloride, backfilled into the tip. The micropipette was baked for 4.5 min at 450°C on a hot plate. H^+^-sensitive cocktail (Fluka 95291, Fluka) was backfilled into the silanized tip and filled up with 0.1 M Na-citrate, pH 6.0. To increase the opening of the electrode-tip, it was beveled with a jet stream of aluminum powder, suspended in H_2_O. The reference electrode was filled with 3 M KCl. Calibration of the electrodes was carried out in oocyte salines with a pH of 7.0 and 6.4. As described previously [37], optimal pH changes were detected when the electrode was located near the inner surface of the plasma membrane. During all measurements, oocytes were clamped to a holding potential of -40 mV using an additional microelectrode, filled with 3 M KCl and connected to an Axoclamp 2B amplifier (Axon Instruments). All experiments were carried out at room temperature (22-25°C). The measurements were stored digitally using custom made PC software, based on the program LabView (National Instruments). The rate of change of the measured [H^+^]_i_ was analyzed by determining the slope of a linear regression fit using OriginPro 8.6 (OriginLab Corporation). Conversion and analysis of the data has been described in detail previously [35].

### Determination of CA catalytic activity via mass spectrometry

Catalytic activity of carbonic anhydrase in *Xenopus* oocytes and cancer cells was determined by monitoring the ^18^O depletion of doubly labelled ^13^C^18^O_2_ through several hydration and dehydration steps of CO_2_ and HCO_3_^-^ at 24°C [38, 39]. The reaction sequence of ^18^O loss from ^13^C^18^O^18^O (m/z = 49) over the intermediate product ^13^C^18^O^16^O (m/z = 47) and the end product ^13^C^16^O^16^O (m/z = 45) was monitored with a quadrupole mass spectrometer (OmniStar GSD 320; Pfeiffer Vacuum, Asslar, Germany). The relative ^18^O enrichment was calculated from the measured 45, 47, and 49 abundance as a function of time according to: log enrichment = log (49×100/(49+47+45)). For the calculation of CA activity, the rate of ^18^O degradation was obtained from the linear slope of the log enrichment over the time, using OriginPro 8.6. The rate was compared with the corresponding rate of the non-catalyzed reaction. Enzyme activity in units (U) was calculated from these two values as defined by Badger and Price [40]. From this definition, one unit corresponds to 100% stimulation of the non-catalyzed ^18^O depletion of doubly labelled ^13^C^18^O_2_. For determination of CA activity in oocytes, batches of 20 native oocytes or 20 oocytes expressing CAIX-WT or CAIX-ΔPG were lysed in oocyte saline (pH 7.0), pipetted into the cuvette and the catalyzed degradation was determined for 10 min. To determine the effect of the antibodies Anti-PG and Anti-CA on CA activity in oocytes, batches of 20 native oocytes or 20 oocytes expressing CAIX-WT were lysed in 100 μl of ice-cold oocyte saline (pH 7.0) and spun down for 10 min at 13,000 g, 4°C to remove yolk and cell debris which could interfere with the binding between CAIX and antibody. This procedure did also lead to the loss of some protein, resulting in a slightly decreased catalytic activity as compared to untreated oocyte lysate. 95 μl of the cleared lysate were transferred to a new centrifuge tube and 5 μl of Anti-PG (100 μg/ml), 5 μl of Anti-CA (100 μg/ml), or 5 μl of oocyte saline were added to the lysate. Lysates were incubated for 2h at 4°C on a rotating shaker before measurement.

### Cultivation of MCF-7 and MDA-MB-231cells

The human breast adenocarcinoma cell lines MCF-7 and MDA-MB-231 were purchased from the German Collection of Microorganisms and Cell Cultures DSMZ, Braunschweig, Germany (DSMZ-No. ACC-115, ACC-732). MCF-7 cells were cultured in RPMI-1640 medium (Sigma-Aldrich, Schnelldorf, Germany), supplemented with 10% fetal bovine serum, 10 mg human insulin, 5 mM glucose, 16 mM NaHCO_3_, 1% penicillin/streptomycin and 2% MEM amino acids solution (Sigma-Aldrich, Schnelldorf, Germany), pH 7.2. MDA-MB-231 cells were cultured in Gibco Leibovitz-L15 medium (Life Technologies GmbH, Darmstadt, Germany), supplemented with 10% fetal calf serum, 5 mM glucose and 1% penicillin/streptomycin, pH 7.4. Both cell lines were incubated at 37°C in 5% CO_2_, 20% O_2_, 75% N_2_ (normoxia) or 5% CO_2_, 1% O_2_, 94% N_2_ (hypoxia, 3 days) in humidified cell culture incubators.

### pH Imaging in MCF-7 and MDA-MB-231 cells

Changes in intracellular pH were measured with an epifluorescence microscope (BX50WI upright microscope, Olympus Deutschland GmbH, Hamburg, Germany; with Polychrome IV epifluorescence unit, Till Photonics GmbH, Munich, Germany). Cells were loaded with 2 μM of the acetoxymethyl ester of 2',7'-biscarboxyethyl-5,6-carboxy fluorescein (BCECF-AM; Life Technologies) for 15 min. BCECF was excited for 5 ms in an interval of 0.2 Hz, at a wavelength of 440 nm followed by 490 nm. The 535 nm fluorescence emission (F) of the two wavelengths’ was monitored through a LUMPlanFI 20x/0.5w objective (Olympus) with a peltier-cooled CCD camera (Till Photonics). The emission strength at 490 nm is inverted proportional to pH while the emission strength at 440 nm remains constant during pH changes, which allows to achieve a pure pH-dependent signal by calculating the ratio F440/F490. The system was calibrated by the use of the K^+^/H^+^-exchanging ionophore nigericin (10 μM; Life Technologies) and the fluorescence signals converted to pH. Data analysis was carried out with the program Clampfit (Molecular Devices, Sunnyvale, California).

Cells were mounted under the microscope in petri dishes (Falcon™ Bacteriological Petri Dishes #351008, Fisher Scientific) and constantly perfused with medium at a rate of 2 ml/min at room temperature. The medium had the following composition: 143 mM NaCl, 5 mM KCl, 2 mM CaCl_2_, 1 mM MgSO_4_, 1 mM Na_2_HPO_4_, 10 mM HEPES, pH 7.2. In CO_2_/HCO_3_^-^- and lactate-containing media, respectively, NaCl was substituted by NaHCO_3_ or Na-lactate in equimolar amounts. To reproducibly measure the rate of lactate-induced acidification, cells were depleted of lactate for at least 15 minutes prior to lactate application.

### Lactate imaging in MCF-7 and MDA-MB-231 cells

MCF-7 and MDA-MB-231 cells were transduced with adenovirus carrying the lactate-sensitive FRET nanosensor *Laconic* (custom made by Vector Biolabs, Philadelphia, USA). Specifications of the FRET sensor have been described in detail previously [41]. For transduction, 1×10^4^ cells were resuspended in 800 μl of serum-free Gibco OptiMEM medium (Life Technologies GmbH, Darmstadt, Germany) and plated on a petri dish (Falcon™ Bacteriological Petri Dishes #351008, Fisher Scientific). Immediately after plating 0.8 μl of virus, containing *Laconic* (4.8×10^10^ PFU) was added to the medium. Cell culture medium was added 4 h after transduction. Cells were incubated overnight under normoxic conditions. Afterwards the transduction process was stopped by exchange of medium and cells were incubated for three days under normoxic and hypoxic conditions, respectively.

All imaging experiments were performed at room temperature with a Zeiss LSM 700 confocal laser scanning microscope. *Laconic* was excited at 405 nm. The fluorescence was split at 508 nm into a <508 nm fraction and a >508 nm fraction. For ratiometric imaging of lactate concentration the <508 nm fraction was divided by the >508 nm fraction. Data analysis was carried out with the program OriginPro 8.6.

Since Laconic has a high affinity for lactate, the sensor is well suited to reliably measure the rate of lactate uptake, but not the rate of lactate efflux (due to the high time constant of lactate release from the sensor the rate of efflux may be underestimated) [18]. Therefore Laconic was only used to measure the influx, but not the efflux of lactate.

### Western blot analysis

Detection of CAIX was performed either with rabbit anti-human CAIX polyclonal antibody (2 μg/ml; NB100-417, Novus Biologicals), for samples of cells treated with anti-CAIX monoclonal antibodies, or with mouse anti-human CAIX monoclonal antibody clone Anti-PG (M75; 0.4 μg/ml). As a loading control, β-tubulin was labelled with anti-β-tubulin mouse monoclonal antibody (2 μg/ml; Clone TUB 2.1 T5201, Sigma-Aldrich). Primary antibodies were labelled with goat anti-rabbit or goat anti-mouse IgG horseradish peroxidase-conjugated secondary antibody (0.2 μg/ml; sc-2004 and sc-2005, Santa Cruz Biotechnology). Quantification of proteins was carried out with the software ImageJ. CAIX protein concentrations were normalized to the concentration of β-tubulin in the same probe.

### Antibody staining of CAIX in *Xenopus* oocytes

*Xenopus* oocytes, coexpressing MCT1 + CAIX-WT, MCT4 + CAIX-WT, MCT1 + CAIX-ΔPG, MCT4 + CAIX-ΔPG, as well as native control oocytes, were fixed for 30 min in 4% paraformaldehyde (PFA) in phosphate-buffered saline (PBS). Oocytes were permeabilized with 100% methanol for 10 min at -20°C and 0.5% Triton X-100. Unspecific binding sites were blocked with 3% bovine serum albumin (BSA) and 1% normal goat serum (NGS) for 2h at room temperature. The cells were incubated with rabbit anti-CAIX, raised against the peptide corresponding to the intracytoplasmic (IC) region of the protein (20 ng/μl) in PBS, supplemented with 1% BSA and 0.1% Triton X-100 overnight at 4°C. Oocytes were washed with PBS and incubated with the secondary antibody (Alexa Fluor 488 goat anti-rabbit IgG, Molecular Probes, Inc., USA). Oocytes were analyzed with a confocal laser scanning microscope (Leica TCS SP5, Leica Microsystems, Wetzlar, Germany) with a 20x objective (HCX-PL-APO-CS-20x/0.7-DRY, Leica Microsystems), using whole oocytes, through which cross sectional optical planes were laid. Because whole oocytes were used, the pictures show only the CAIX fraction in the plasma membrane and not the cytosolic protein fraction.

### Measurement of cell proliferation

MCF-7 and MDA-MB-231 cells were incubated at 1% O_2_ in the presence of Anti-PG (5 μg/ml) and Anti-CA (5 μg/ml), respectively, or without antibody. Cells were plated at a density of ≥ 1×10^4^ cells/ml and incubated for 0-3 days under hypoxic conditions in a cell culture incubator. Cells were counted each day using a phase contrast microscope and placed back into the incubator after counting. Five images were taken from each dish at random locations. After day 3 cells were fixed with Roti-Histofix^®^ (Roth). Nuclei were stained with the fluorescent dye Hoechst 33342 (10 μM; Life technologies) and images were taken with a confocal laser scanning microscope (Zeiss LSM 700). The number of cells per image was counted using the software ImageJ.

### Measurement of cell migration

MCF-7 and MDA-MB-231 cells were incubated under normoxic conditions until they reached confluence. After confluence was reached, cells were incubated under normoxic or hypoxic conditions in the presence of Anti-PG (5 μg/ml) and Anti-CA (5 μg/ml), respectively, or without antibody. A scratch was made with the tip of a pipette through the confluent culture. Five images were taken from the scratch area over 3 days using a phase contrast microscope with camera (Olympus). After day 3 cells were fixed with Roti-Histofix^®^ (Roth). Nuclei were stained with the fluorescent dye Hoechst (1:1000; Life technologies) and images were taken with a confocal laser scanning microscope (Zeiss LSM 700). The area of scratch per image was analyzed using the software ImageJ. Data were corrected for cell proliferation by using the formula

F_corr_ = F_0_ − (P ∙ F_cell_)

with F_corr_ = corrected cell-free area

   F_0_ = measured, not corrected cell-free area

   P = rate of cell proliferation, as measured by the proliferation assay

   F_cell_ = average surface area of one cell

The resulting data were normalized to the values at the beginning of the experiment and as per cent values against the time.

### Calculation and statistics

Statistical values are presented as means ±standard error of the mean (SEM). For calculation of significance in differences between two sets of data, Student's t-test, following a Shapiro-Wilk test for normal distribution, was used. For calculation of significance in difference between more than two sets of data one way analysis of variance (ANOVA) was carried out, followed by means comparison using either Scheffé or Bonferroni test, depending on whether datasets show homogeneity of variance or not. Homogeneity of variance was assessed using Levene's test. All statistical tests were carried out with OriginPro 8.6. In the figures shown, a significance level of p ≤ 0.05 is marked with ^*^, p ≤ 0.01 with ^**^ and p ≤ 0.001 with ^***^.

## Abbreviations

Ab: antibody
BCECF-AM: 2',7'-biscarboxyethyl-5,6 carboxy fluorescein - acetoxymethyl ester
CAIX: carbonic anhydrase isoform IX
HEPES: 4-(2-hydroxyethyl)-1-piperazineethanesulfonic acid
LE: log enrichment
MCF-7: Michigan Cancer Foundation – 7 (cell line)
MCT: monocarboxylate transporter
MDA-MB-231: M.D. Anderson - metastatic breast – 231
PG: proteoglycan-like
WT: wild type
